# Genomic insights on the ethno-history of the Maya and the ‘Ladinos’ from Guatemala

**DOI:** 10.1186/s12864-015-1339-1

**Published:** 2015-02-25

**Authors:** Jens Söchtig, Vanesa Álvarez-Iglesias, Ana Mosquera-Miguel, Miguel Gelabert-Besada, Alberto Gómez-Carballa, Antonio Salas

**Affiliations:** Unidade de Xenética, Departamento de Anatomía Patolóxica e Ciencias Forenses, and Instituto de Ciencias Forenses, Facultade de Medicina, Universidade de Santiago de Compostela, CP 15872 Galicia, Spain

**Keywords:** Mesoamerica, Guatemala, Maya, Ladino, mtDNA, Y-chromosome, AIMs, Autosomal SNPs, Phylogeography

## Abstract

**Background:**

Guatemala is a multiethnic and multilingual country located in Central America. The main population groups separate ‘Ladinos’ (mixed Native American-African-Spanish), and Native indigenous people of Maya descent. Among the present-day Guatemalan Maya, there are more than 20 different ethnic groups separated by different languages and cultures. Genetic variation of these communities still remains largely unexplored. The principal aim of this study is to explore the genetic variability of the Maya and ‘Ladinos’ from Guatemala by means of uniparental and ancestry informative markers (AIMs).

**Results:**

Analyses of uniparental genetic markers indicate that Maya have a dominant Native American ancestry (mitochondrial DNA [mtDNA]: 100%; Y-chromosome: 94%). ‘Ladino’, however, show a clear gender-bias as indicated by the large European ancestry observed in the Y-chromosome (75%) compared to the mtDNA (0%). Autosomal polymorphisms (AIMs) also mirror this marked gender-bias: (*i*) Native American ancestry: 92% for the Maya *vs*. 55% for the ‘Ladino’, and (*ii*) European ancestry: 8% for the Maya *vs*. 41% for the ‘Ladino’. In addition, the impact of the Trans-Atlantic slave trade on the present-day Guatemalan population is very low (and only occurs in the ‘Ladino’; mtDNA: 9%; AIMs: 4%), in part mirroring the fact that Guatemala has a predominant orientation to the Pacific Ocean instead of a Caribbean one. Sequencing of entire Guatemalan mitogenomes has led to improved Native American phylogeny *via* the addition of new haplogroups that are mainly observed in Mesoamerica and/or the North of South America.

**Conclusions:**

The data reveal the existence of a fluid gene flow in the Mesoamerican area and a predominant unidirectional flow towards South America, most likely occurring during the Pre-Classic (1800 BC-200 AD) and the Classic (200–1000 AD) Eras of the Mesoamerican chronology, coinciding with development of the most distinctive and advanced Mesoamerican civilization, the Maya. Phylogenetic features of mtDNA data also suggest a demographic scenario that is compatible with moderate local endogamy and isolation in the Maya combined with episodes of gene exchange between ethnic groups, suggesting an ethno-genesis in the Guatemalan Maya that is recent and supported on a cultural rather than a biological basis.

**Electronic supplementary material:**

The online version of this article (doi:10.1186/s12864-015-1339-1) contains supplementary material, which is available to authorized users.

## Background

**Figure 1:**
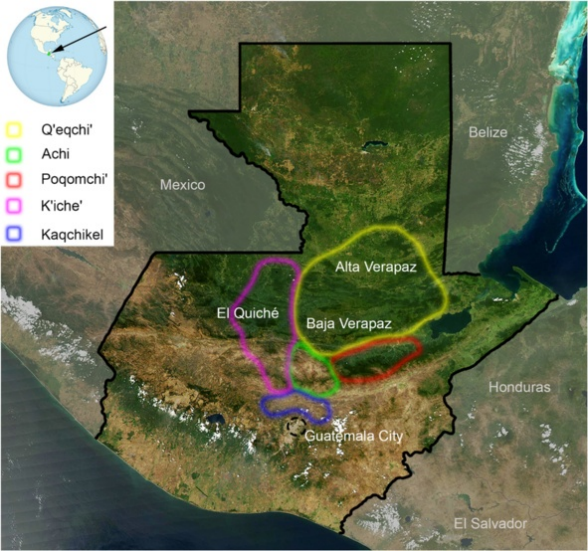
**Map of the Republic of Guatemala.** Colored shapes indicate the areas were the Mayans analyzed in the present study live at present. Most of the samples were recruited in the departments of Alta and Baja Verapaz, El Quiché, and the Capital city of Guatemala. The satellite image was taken from the NASA Visible Earth catalogue (http://visibleearth.nasa.gov/).

The Republic of Guatemala is located in Central America, bordering the Pacific Ocean, between El Salvador and Mexico, and the Caribbean Sea between Honduras and Belize (Figure 1). Guatemala is a multiethnic, multicultural and multilingual country with an estimated population of about 14.7 million people in 2011 (according to the *Instituto Nacional de Estadística of Guatemala*; INE; http://www.ine.gob.gt).

The main populations are the ‘Ladinos’ (~60%), a term used in Central America (deriving from ‘latino’), and especially in Guatemala, to refer to a mix of Native American and Spanish (and eventually of Africans), and the Maya or ‘Indígena’ (~40%), that constitutes the second most important group in the country. The ‘Ladino’ population of Guatemala is officially recognized by the *Ministerio de Educación* (MINEDUC; http://www.mineduc.gob.gt/) as a heterogeneous population, which expresses itself in Spanish as a maternal language and possesses specific cultural traits of ‘Hispanic’ origin mixed with indigenous cultural elements. Already in 1690, the chronicler Francisco Antonio de Fuentes y Guzmán described the ‘Ladinos’ as ‘mestizos, mulatos and negros’. There is extensive historical documentation indicating trend in Guatemala to marriages between different ethnic groups [1]. Although the demographic impact of Europeans in Guatemala is difficult to quantify, it is estimated that in the beginning of the XVII century, the indigenous population surviving in Guatemala (and other Central American countries such as El Salvador) constituted only 10% of the total population living in the region before the arrival of Europeans [1]. The impact of the slave trade in Guatemala is also difficult to estimate. Some documentation indicate that in 1773, the population of Santiago de Los Caballeros de la Antigua Guatemala (‘the capital of Centro America’) had 30.000 people, and about 36% of them were ‘mulatos’ (admixed between Africans and Europeans or Natives), and in 1782, the ‘mulatos’ constituted 32% of a total of 13.000 inhabitants in the city of Nueva Guatemala de la Asunción [1] (the present Capital city of Guatemala). These figures could indirectly indicate the existence of an important amount of slaves in the regions. In contrast, the Trans-Atlantic Slave Trade Database (http://www.slavevoyages.org/) shows that only a few hundred slaves disembarked directly in Guatemala. However, the arrival of important amounts of slaves from other neighboring countries that were more connected to the slave trade (such as Honduras and Belize) cannot be disregarded.

Among the present-day Maya from Guatemala, there are more than 20 different ethnic groups including the K’iche’ (9.1%), Kaqchikel (8.4%), Mam (7.9%), Q’eqchi’ (6.3%), and minority groups such as Achi, Akatek, Chuj, Ixil, Jakaltek, Poqomam, Poqomchi’, Q’anjob’al, Tz’utujil, Uspantek, etc. (altogether 8.6%; according to the 2001 census). Ethnicity names usually refer to the indigenous language spoken by the group members. Although Spanish is the official language in Guatemala today, there are 23 officially recognized Native American languages. It is not uncommon that people from one region of Guatemala do not understand the language of a neighboring region. For most Mayan inhabitants, Spanish is a second language, and many Maya do not speak Spanish at all in some areas of the country. Today, the largest proportion of the Guatemalan Maya population lives in the highlands (where the majority of the studied samples of the present study have been taken), but there are also inhabitants in other rural areas, such as El Quiché department. Other minority Native American groups in Guatemala are the Garifuna and Xinka (0.1%).

The Maya constituted vast kingships during a long period over the Mesoamerican landscape (a term describing Mexico and Central America within which a number of pre-Columbian societies flourished before the Spanish colonization in the XVI century [2]), a reign that lasted for about three thousand years (kya) and was one of the most advanced civilizations within the New World. The first concrete traces of the Mayan civilization (dating back to the Pre-Classic period around 1,800 BCE) were found in the *Mirador Basin* of the northern department of Petén (Guatemala), though some settlements are thought to be over 6 kya old [3]. The *Mirador Basin* is part of a larger region (known as the Guatemala’s Maya Biosphere Reserve) that overall is considered to be the cradle of ancient Maya civilization (>175 archaeological sites).

According to current knowledge, a single language existed among the earliest Maya. This Proto-Mayan is thought to have been spoken at least ~4 kya ago and may be the common ancestor of all modern Mayan languages today, as well as the Classic Maya languages documented in the hieroglyphic inscriptions [4]. Reconstructive and descriptive linguistic studies of ancient Proto-Mayan target the Guatemalan highlands as the birthplace of this ancestral language [5]. Because of the isolation of Maya posted by vast distances and the ecological diversity of their territories, regional conflicts, sporadic migrations, and ever-changing political systems, their language has had acquired many pronunciations, and over time, those dialects have spawned new languages [6].

During the Pre-Classic Period (2000 BCE–250 CE), a great linguistic diversity developed, comprising 16 language families. Unlike other scattered populations of Mesoamerica, the Maya were centered in one geographical area covering the entire Yucatan Peninsula and modern-day Guatemala; Belize and parts of East Mexico; and the western region of El Salvador and Honduras.

It was during the Classic period (AD 250–900) that the Maya civilization reached the peak of its power and influence and it was one of the most dominant indigenous societies of Mesoamerica. During this period, the Mayan civilization had become a complex and dynamic entity of independent city-states undergoing a series of population expansions and contractions [5,7,8]. These fluctuations may reflect episodes of migration at various times during the Classic period. By the Late Classic period (AD 600–900), much of the Maya region was organized into two competing “super-states,” headed by the hegemonic powers of *Tikal* and *Calakmul* [2,9]. By the terminal Classic period, massive declines in population size led to the abandonment of many Maya territories. The reason for this subsidence remains largely unknown, although theories invoke environmental over-exploitation with all of its consequences as well as constant warfare in a landscape divided among numerous competing city-states as the main reasons.

In the Post-Classic (AD 900–1,500) period, the fragmentation process led to a fusion of Maya settlements from the southern Yucatán highlands into the regionally dominating K’iche’ and later Kaqchikel states [5]. Constant strains within the Maya region and with non-Mayan groups (Aztecs and Toltec of Mexico) led to the final collapse of the civilization prior to the arrival of the Spanish [5].

Nowadays, Maya descendants occupy the territories of Mexico, Guatemala, Belize, El Salvador, and Honduras. Maya people mostly follow their traditional way of life, including costumes, indigenous languages, and religious ceremonies. One of their most remarkable cultural traits is the faithful count of days according to the Maya calendar.

Historic evidence based on patterns of material culture (ceramics), as well as geographic variability in agricultural practices and socio-political structures suggest a degree of regional isolation which leads to an explanation of the Classic Maya population structure as a model of isolation by distance (IBD). This model describes the tendency of populations that are geographically closer to be more similar than populations that are further apart [10]. Such a model would consider ancient Maya as relatively non-mobile population groups and inter-population gene flow restricted to neighboring sites. Over their millenary history and given the great distances between their communities, strengthened by separation through geographical barriers, warfare, and their political system of independent city-states, it may be expected that the Maya would have diverged into several distinct populations. Contradictory evidence (mostly inscription based) shows however long-distance trade, elite visits and marriage and intercity conflicts with captive-taking, as well as the mobility of general populations. On the other hand, Mayan art, architecture and rituals suggest a high degree of cohesiveness throughout their domain. Overall, there is enough evidence indicating that certain gene flow occurred across the entire Maya area during the Classic period.

While both a rich archaeological record and hieroglyphic dataset led to a better understanding of the Classic Maya population history compared to most other ancient Native American cultures [2], biological investigations of ancient Guatemala population history are mainly limited to osteology [11] and dental studies [12,13]. These studies arrived however to contradictory findings. Thus, dental morphology examinations found evidence for biological discontinuity at *Seibal* (Petén) between the Late and Terminal Classic periods [14]. Using the same approach, it could be shown that skeletons of *Jaina* (Yucatán, Mexico) demonstrated a stronger affiliation to the Petén site than they did to nearby *Chichen Itza* in Mexico [15]. In addition, distinct regional clustering could not be found by odontometric comparison of individuals from five Maya sites in the Yucatán Peninsula [16]; these studies suggested that extensive gene flow dominated Classic Maya population structure with genetic exchange not only between intrazonal neighbors, but at least partially between long distances. Analyses of odontometric variation, as carried out by Sherer et al. [13], suggested the existence of important gene flow during the Classic period; these authors found an overall *F*_ST_ value of 0.018, indicating little among-group variability for the Classic Maya sites tested. Furthermore, chemical evidence by isotope analyses points in the same direction, suggesting that migration of both elites and general populations was greatest during the Early Classic period [17,18].

Analysis of uniparental DNA markers (especially mitochondrial DNA [mtDNA] variation) and autosomal DNA markers has been widely used to explore demographic patterns throughout the Americas [19-36]. However, genetic analyses on present-day populations from Guatemala are limited to only a few studies. The study by Ibarra-Rivera et al. [37] on autosomal STRs (typically used in forensic genetics) revealed that Maya showed fewer alleles and decreased levels of heterozygosity compared to Asians, Europeans, North And South Americans, and even non-Mayan Mesoamericans. This study also supplied evidence that the Guatemalan Mayan groups appeared less genetically variable than their Yucatan counterparts, which supports the thesis that the Guatemalan Maya have experienced less gene flow than the Maya from the Mexican Peninsula [38]. Note that increased allelic diversity is expected in the Yucatan’s plateau because of the greater accessibility afforded by a lack of major geographical barriers [5,39]. The limited genetic diversity of the Maya was indicative of isolation, founder effects, bottlenecks, limited gene flow from neighboring non-Mayan peoples, and/or possible inbreeding. Overall, the results of this study suggested that though some genetic variability exists between Mayan groups, there is a higher degree of homogeneity between them than when compared with other Mesoamerican populations. This led to the presumption that distinct Mayan settlements did not evolve in isolation from one another. In fact, the data indicate that the cultural similarities shared by the Maya are reflected in their genetic profiles and are not merely a result of geographic and/or cultural interactions [37]. Furthermore, data on HLA genes [40], and polymorphic *Alu* insertion (PAI) *loci* [38] in the actual Guatemalan population were also generated. When comparing HLA allelic frequencies in the Maya from Guatemala with other worldwide populations, the Maya were found to differ genetically from the Mixe and Oaxacan-Mexican Native Americans and to show a close relationship to the Arhuacs, Kogi, and Arsario tribes of the Caribbean. Based on the *Alu* insertion polymorphisms, two geographically adjacent Mayan populations from the Guatemalan highlands (K’iche’ and Kaqchikel) were found to be more similar to each other than to populations from Yucatán. Other studies reported autosomal data on the distribution of standard autosomal STRs in the actual ‘Ladino’ as in the Maya population of Guatemala [37,41] and Mexico [42].

Genetic data on uniparental markers of Guatemalans are also very limited. Nonetheless, forensic genetics played a key role in the investigation of the Maya homicide during Guatemala’s 30-year-long Civil War by the *Fundación de Antropología Forense de Guatemala* (FAFG; http://www.fafg.org/) [43]. Almost 20 years ago, DNA extraction from bones and sequencing of the mtDNA HVS-I was carried out after the exhumation of a 10-year-old clandestine mass grave. The study involved samples collected in a Quiché Indian village located close to the provincial capital of Santa Cruz de Quiché [44], and lead to the determination of 16 different mtDNA haplotypes. Recently, a study on 17 Y-STR *loci* in a set of 115 ‘Mestizo’ and 110 Maya males allowed further insight into the actual level of genetic variability and population structure of the populations of the country [45]. The authors clearly identified Guatemalans as predominantly Native Americans and detected a population sub-structure differentiating ‘Mestizos’ from Mayans to certain extent.

Finally, studies on Mayan ancient DNA were not successful due to the poor preservation of Maya skeletons [46].

The aim of the present study is to shed light on the demographic and the ethno-history of the Guatemalan indigenous populations and characterize the admixture proportions of Mayas and ‘Ladinos’ of Guatemala by means of uniparental (Y-chromosome and mtDNA) and Ancestry Informative Markers (AIMs).

## Results and discussion

### Mitochondrial DNA control region variation

Additional file 1 reports the full mtDNA control region plus mtSNP haplotypes obtained in the present study, and provides the haplogroup classification according to the level of phylogenetic resolution obtained.

**Figure 2:**
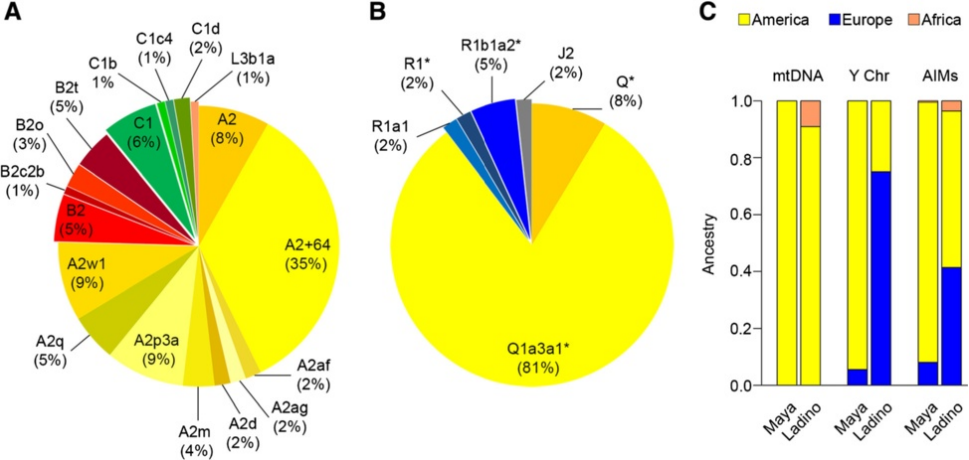
**Distribution of Native American haplogroup frequencies and average continental ancestry in the Guatemalan sample sets.** The pie charts represent the distribution of mitochondrial **(A)** and Y chromosomal haplogroup **(B)** frequencies. In (B), R1b1a2* refers to R1b1a2*(×R1b1a2a1a1, R1b1a2a1a2a1b1a, R1b1a2a1a2b, R1b1a2a1a2c1a1a1), and Q* to Q*(×Q1a3a1), R1* to (×R1a, R1b1), and Q1a3a1* to Q1a3a1*(×Q1a3a1a-c). The bar chart **(C)** shows the average continental ancestry according to mtDNA, Y chromosomal and AIM-InDel data for the ‘Ladino’ and Maya population. Percentages of ancestry on the autosomal markers were obtained from the admixture analysis (and the optimal value *k* = 3). Percentages in the pie charts were obtained from Additional files 1 and 4 and rounded-up.

Guatemala shows a main mtDNA Native American component (99%). All haplotypes, except one, can be classified into one of the main Native American mtDNA haplogroups: A2 (75%), B2 (14%), and C1 (10%), (Figure 2A). Within A2, the most common sub-haplogroups are A2 + C64T (35%), A2p (9%) and A2w1 (9%). Within B2, the most common sub-haplogroup is B2t, accounting for 5% of the total mtDNAs.

**Figure 3:**
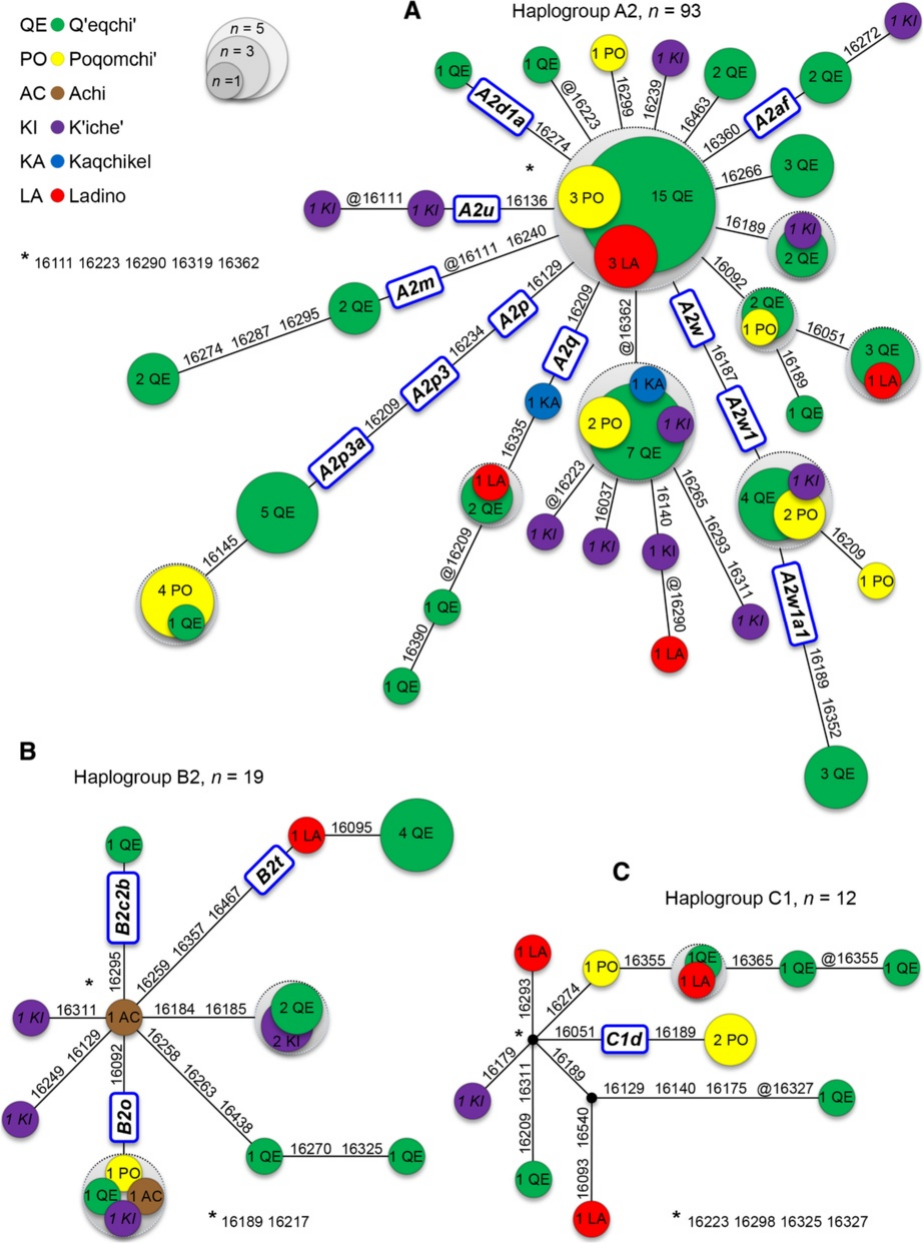
**Phylogenetic network of mtDNA HVS-I sequences belonging to Native American haplogroup A2 (A), haplogroup B2 (B) and haplogroup C1 (C).** Circle sizes are proportional to the haplotype frequencies. The 16 K’iche’ HVS-I haplotypes reported by Boles et al. [44] are indicated by letters in italics.

All Guatemalan Native American profiles were resolved into a maximum parsimony network (Figure 3). The 16 K’iche’ HVS-I haplotypes reported by Boles et al. [44] were also included in these networks (see also Additional file 1). The phylogeny of haplogroup A2, the most common haplogroup in the Maya and ‘Ladino’ (Figure 3A), is mainly star-like, but it also shows some derived branches containing haplotypes that appear overrepresented, and as is the case for branches A2 + @T16362C, A2 + T16092C, A2q, A2p3a, etc. (Figure 3A). The most common control region haplotype corresponds to the root of haplogroup A2. One interesting feature of the network of haplogroup A2 is the large proportion of haplotypes that are shared between the different ethnic groups. This is particularly notable for those better represented our sample, that is, Q’eqchi’, Poqomchi’ and K’iche’. In other words, there is no particular clade that is overrepresented in one of the Maya groups.

Although the sample size is lower than for A2, haplogroups B2 and C1 show similar phylogenetic patterns (Figure 3B and C, respectively).

As expected, the admixed population of ‘Ladino’ follows the same pattern as the Maya; their haplotypes are scattered through the different branches of the A2, B2, and C1 phylogeny.

A phylogeographic connection between Guatemala and North and South America is evident not only for the most common haplotypes but also when examining singular haplotypes. For instance, A2 haplotypes #GT06 and #LaTinta_20 (Additional file 1), characterized by a reversion at T16362C and T16140C on top of the basal haplotype, is uncommon in North America, but appears in Mexico [31] and in the North of South America, such as Bolivia [47], Peru [48], etc. A similar distribution has the haplotype root of A2 + C16266T. The lineage B2o, observed in four Guatemalans, is also found all over the American continent, from Native North Americans [49] to the Southern Cone [27].

However, a large number of other haplotypes have a clear predominant or even exclusive distribution in Mesoamerica. Some examples are: (i) A2u members appear mainly in Panama [29], El Salvador [26], and Mexico [31,50], (ii) root of A2 + A16299G is also very frequent in El Salvador [26], Costa Rica [51], Nicaragua [52], and Panama [29], and (iii) root of A2 + A16274G (haplogroup A2d1a) appearing in Panama [29,53], and Mexico [54].

Additional searches of all of the profiles included in Figure 3 were carried out in other databases. For instance, in haplotype queries performed in EMPOP, and excluding the so-called “admixed” individuals from the USA (composed in part by individuals coming from different American countries, e.g. Mexico), indicated that the great majority of haplotypes were found almost exclusively in Mesoamerica (~61% in Mexico and ~33% in Guatemala).

Some of these and other phylogeographic features became even clearer when entire genomes were examined (see next section).

In the Guatemalan samples, there was only one haplotype (#GT24) of recent Sub-Saharan ancestry belonging to L3b1a. This haplotype could have arrived in Guatemala at the times of the Trans-Atlantic slave trade [55,56], although this haplogroup is more common in East Africa than in West or West-Central Africa [57,58]. It is important to note that this donor does not show any notable African autosomal ancestry (AFR: 0.3%, EUR: 64.6%, AME: 35.1%; see below) and describes itself as ‘Ladino’. This suggests that the carrier of this African lineage is not a very recent arrival into Guatemala.

### Phylogeography of Maya mitogenomes and Time of the Most Recent Common Ancestor (TMRCA)

Ten out of the twelve mitogenomes analyzed belong to haplogroup A2, while two belong to haplogroup B2 (Additional file 2). The mitogenomes obtained allowed improved resolution of the mtDNA phylogeny within these Native American haplogroups. In particular, we have defined eight new clades, namely A2w1a, A2w1b, A2w2, A2w3, A2w4, A2p3, A2ar, and B2t1 (together with other minor sub-clades).

**Figure 4:**
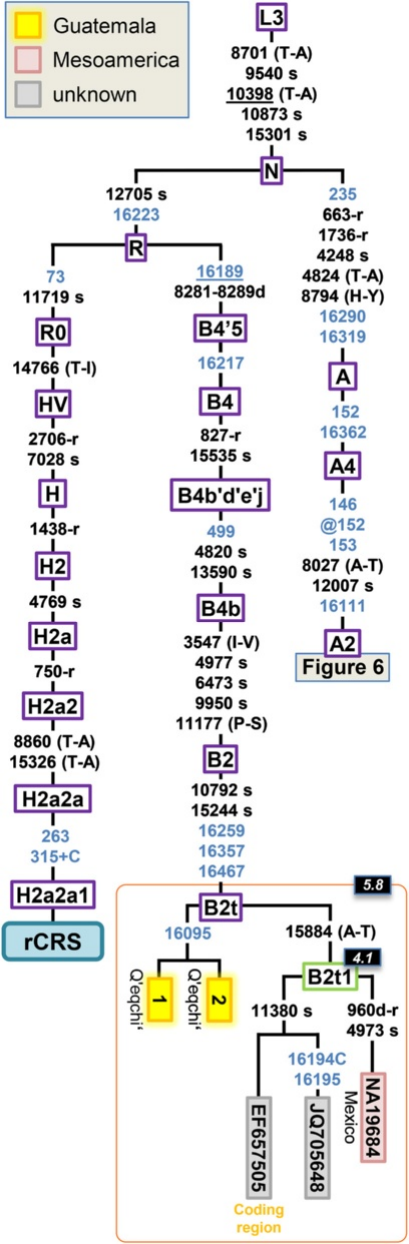
**Maximum parsimony tree of Guatemalan B2 mitogenomes.** The position of the rCRS is indicated for reading off sequence motifs. Mutational changes are shown along branches (in blue are those falling in the control region); mutations are transitions unless a suffix A, C, G, or T indicates a transversion. Other possible suffixes are: insertions (+), synonymous substitutions (s), mutations changes occurring at tRNAs (−t), mutational changes occurring at rRNAs (−r), non-coding variants located in the mtDNA coding region (−nc), and amino acid replacements indicated in round brackets. A back mutation is represented with the prefix “@” (a double “@” would indicate a double recurrent mutation), whereas an underlined mutation represents a recurrent mutation in the phylogeny represented in the figure. As usual, variants at positions A16182C, A16183C, variation around position 310 and length or point heteroplasmies were not considered for the phylogenetic reconstruction. Maximum likelihood (ML) coalescence ages (Table 1) are indicated in the top left corner of each haplogroup label (black background boxes). Green boxes containing haplogroup labels indicate new clades determined in the present study with respect to Phylotree. Coding region segments were not used for estimates of the TMRCA.

Two Guatemalan B2 haplotypes allowed the topology of the haplogroup B2t branch to be re-defined with respect to the current Build 16 version of Phylotree (Figure 4). There are four complete genomes belonging to B2t available; all of them share the sequence motif A10792G-A15244G-C16259T-T16357C-C16467T. The two Maya mitogenomes were found in two Q’eqchi’; these haplotypes are identical and share the transition C16095T. The other two mitogenomes (one sampled in Mexico and the other of unknown origin) and one semi-complete genome (EF657505; coding region) share the transition G15884A and determine the sub-lineage B2t1 (Figure 4). A search of the characteristic HVS-I motif of B2t in the literature (excluding substitution C16467T because it is absent in most control region datasets) reveals that this is a minor haplogroup in America; however, it is present at moderate frequency (5%) in the Nahua, in ‘Mestizos’ from Mexico [31,50] and in an ‘Hispanic’ dataset from USA [59]. Further search of B2t control region profiles in EMPOP reveals the relevant presence of B2t members in Mexico, most of them in the Zoque, which is an ethnic group inhabiting neighboring regions (Chiapas) to Guatemala. There are also a few B2t members classified as ‘admixed’ individuals from the USA (which could constitute recent arrivals from Mexico). B2t therefore has a dominant Mesoamerican distribution. The estimated coalescence age of B2t and B2t1 is 5.8 and 4.1 kya, respectively (Table 1).

**Table 1:** **Haplogroup coalescence time estimates based on ML on mitogenomes**

**Haplogroup**	***N***	**Mean (kya)**	**95% CI (kya)**	**ML distance**	**SD**
A2ar	3	12.22	5.15-19.58	4.59	1.33
A2p	13	8.81	4.12-13.64	3.34	0.90
A2p3	6	3.23	0-7.66	1.24	0.85
A2p3a	5	1.46	0-4.37	0.57	0.57
A2p1	4	3.74	0.29-7.27	1.44	0.68
A2p1b	2	2.03	0-4.61	0.79	0.50
A2p1a	2	1.29	0-3.75	0.50	0.48
A2w	33	9.88	6.99-12.83	3.73	0.55
A2w1	8	9.35	6.46-12.28	3.54	0.55
A2w1a	6	8.58	5.59-11.62	3.25	0.57
A2w1a1	5	7.93	4.84-11.07	3.01	0.59
A2w1b	2	1.52	0-4.09	0.59	0.50
A2w1a1a	3	1.58	0-3.70	0.61	0.41
A2w3	2	3.51	0.62-6.46	1.35	0.57
A2w2	4	6.70	3.41-10.05	2.55	0.63
A2w4	2	7.11	3.47-10.82	2.71	0.70
B2t	4	5.82	0.22-11.62	2.23	1.09
B2t1	2	4.09	0-8.66	1.57	0.87

**Figure 5:**
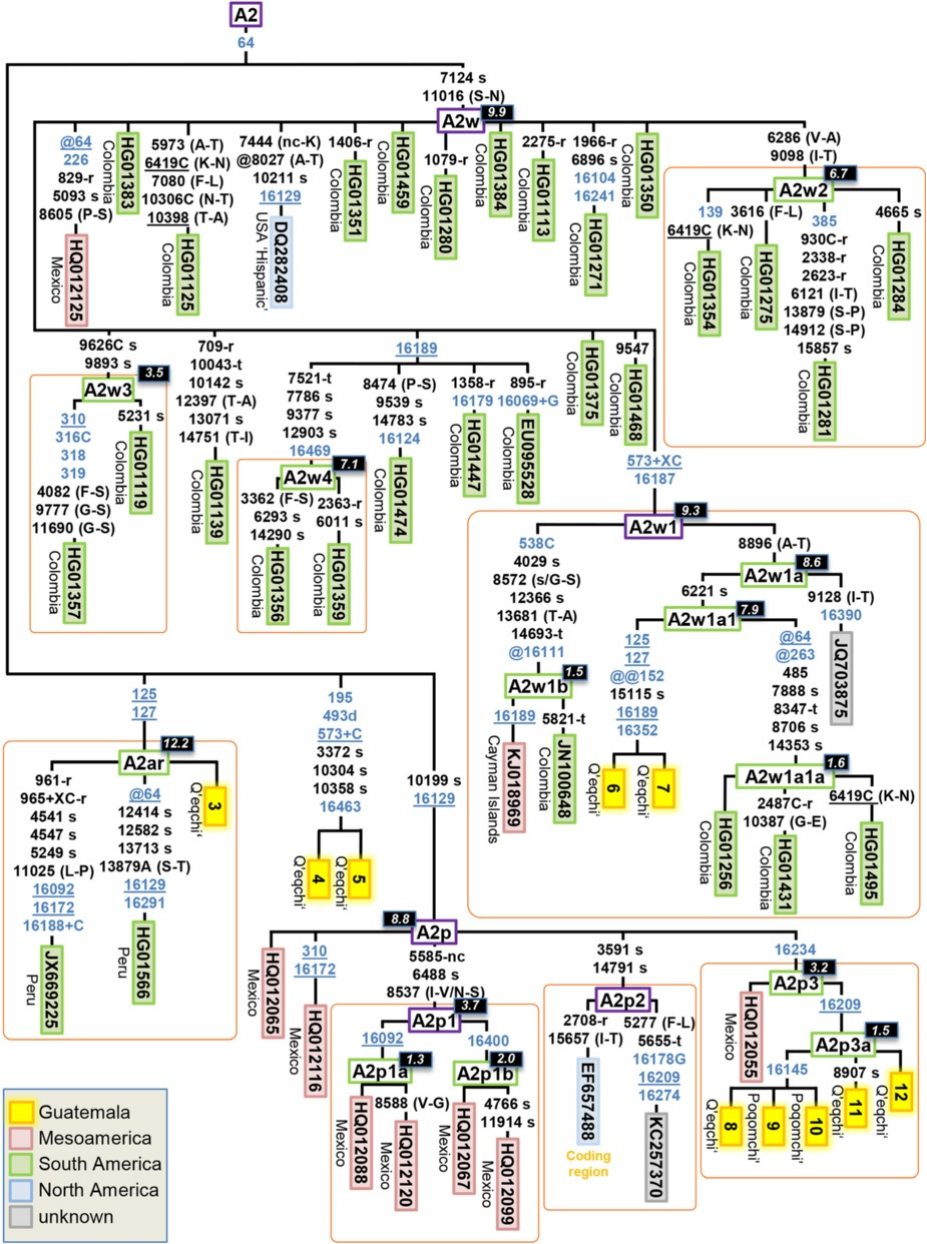
**Maximum parsimony tree of Guatemalan A2 mitogenomes.** See legend of Figure 4 for more details.

Estimate of the time (in kya) to the most recent common ancestor (TMRCA) of each cluster, using evolutionary rate estimates in Soares et al. [60]. First summand (*N*) refers to the complete mtDNA sequences displayed in Figures 4 and 5.

Ten Maya mitogenomes could be classified within different sub-branches of haplogroup A2 (Figure 5). Five of these mitogenomes carry the very stable diagnostic coding region variant C10199T (one mutational hit in Phylotree and 0 in [60]), and therefore belong to haplogroup A2p. Figure 5 shows the updated topology for A2p, which is reconstructed on the basis of 13 mitogenomes and one coding region segment (EF657488). A2p, as a whole, can be dated in 8.8 kya (Table 1). Seven out of these 14 mtDNAs were sampled in Mexico and five in Guatemala (there is no geographic information for the other two; both belong to sub-clade A2p2). Two pairs of mitogenomes from Mexico allow two sub-branches of haplogroup A2p1 (A2p motif + G5585A-T6488C-A8537G) to be determined: A2p1a (A2p1 motif + T16092C) and A2p1b (A2p1 motif + C16400T). A2p1 is a recent clade with an estimated divergence age of 3.7 kya, while A2p1a and A2p1b are 1.3 and 2.0 kya old, respectively. The five Guatemalan samples belong, together with one Mexican haplotype (HQ012055), to haplogroup A2p3 (A2p motif + C16234T); however, all of the Mayan sequences share the substitution T16209C, thus determining a new sub-branch, A2p3a. A2p3a is also relatively new, with an estimated age of 1.5 kya. By searching the control region motif of A2p in the EMPOP dataset, A2p, as a whole, appears mainly in South America (Colombia and Venezuela). A few members of A2p3 can be found in control region databases in Mexico [31], or even sporadically in the North of South America (Venezuela [61]), but the Maya clade A2p3a seems to be basically restricted to the Guatemalan territory.

The sequence motif A7124G-T1101C defines haplogroup A2w, and its topology was determined by 32 mitogenomes, a large number of them analyzed within The 1000 Genome Project in a Colombian sample set (Figure 5). Thus, 27 A2w mitogenomes appeared in Colombia, two in Guatemala, one in Mexico, and one in a ‘Hispanic’ population from the USA (there is an additional mitogenome of unknown origin). In Phylotree, there is only one sub-lineage determined within this haplogroup, namely, A2w1, with the diagnostic motif 573.XC-C16187T. We describe here three additional branches: A2w2, A2w3, and A2w4. The two A2w Guatemalan profiles match entirely and were found in the Q’eqchi’; they share six transitions and belong to the sub-clade A2w1a1. The general topology of A2w is far to be star-like (as measured by the star-likeness index, Additional file 3), and its estimated age using the average distance to the root is paradoxically larger than the age of the entire A2w. This indirectly denotes that sampling of mitogenomes belonging to this sub-clade is probably sub-optimal (dominated by haplogroup members mainly from Colombia). Confirmatory evidence comes from the fact that a search of the control region motif of A2w1a reveals the presence of this haplogroup, mainly all across the Mesoamerican territory, e.g. Panama [62], Costa Rica [51], Nicaragua [52], El Salvador [26], the Garifunas (and Chocó from Caribbean Colombia) [63]. Additional haplotype searches in EMPOP indicate further matches in Honduras and Guatemala (as well as some admixed individual in the USA). Therefore, A2w1 has a wide Mesoamerican distribution but is most likely very prevalent in many South American locations apart from Colombia. Unfortunately, most of the A2w1 sub-branches are not searchable through control region motifs (Figure 5). TMRCA of A2w, as estimated from maximum likelihood (ML), is 9.9 kya; A2w1 would be its oldest sub-clade (9.3 kya). A2w3 is much younger (3.5 kya), as there are some minor sub-clades such as A2w1b (1.5 kya) or A2w1a1a (1.6 kya). The clade containing the two Guatemalan sequences, A2w1a1, is 7.9 kya.

The two HVS-I variants T125C-T127C alone determine haplogroup A2ar. It is important to note that this seeming distinctive sequence motif occurs independently several times in the worldwide phylogeny (e.g. D4l2, L0d2d, M12a1; Phylotree). There are three mitogenomes in A2ar, curiously, two of them sampled in Peru, and one in a Q’eqchi’ individual. This suggests that this minor clade has a Mesoamerican and South American distribution. A2ar seems to be an old sub-clade (12.2 kya) within the phylogeny of A2 (Table 1).

There are two additional A2 Q’eqchi’ individuals sharing exactly the same variants (#4 and #5 in Figure 5). The control region motif of this branch is rare; the most closely related mtDNA in the Americas is a Colla individual from Argentina [64].

### Demographic patterns of the Maya as inferred from mtDNA data

The phylogeny of mtDNA control region haplotypes suggests a complex demographic history in Guatemala as a result of the superposition of different demographic events. The control region network mirrors a main star-like topology, most likely indicating the existence of a recent demographic expansion in the region. This expansion could perfectly fit with the growth of the main Maya centers during the Classic period, about 1.8 kya ago. Superposed to this star-like phylogeny are some deep branches that seem to signal an underlying ancient, more stationary demographic history (which is more clearly revealed by analysis of the complete mitogenomes). The presence of some derived haplotypes from the root occurring at a relatively high frequency reflects the existence of founder events in the different ethnic groups or relative isolation. Furthermore, the presence of identical haplotypes in the analyzed mitogenomes adds further support to the existence of moderate isolation of Maya into relatively small consanguineous groups. However, gene flow between these isolated groups also occurred in Guatemala, as testified by the existence of many haplotypes shared between different Maya groups [19,20,33].

Analysis of mitogenomes reveals a few interesting features of the past Guatemalan demography (Figures 4 and 5). Some haplogroups, for example A2ar and A2w (and some of its sub-clades), date back to the Paleo-Indian period in the chronology of Mesoamericans. These clades appeared about 10–12 kya, and could have arisen in Mesoamerica or in the limits with North America soon after the initial colonization of the Americas; they could have moved in successive colonization waves as far as to the southern continental cone (as already reported for other clades [19,20] or based on autosomal markers [33]). When examining the combined picture provided by the mitogenomes and the control region data, this is supported by the high prevalence of these clades in Mesoamerica and in South America, but only sporadically in admixed individuals from North America.

Some mtDNA clades examined in the present study provide clear evidence for the existence of an important gene flow occurring between the territories of Mesoamerica and South America during the Pre-Classic Era about 4 kya, connecting Mexico, Central American populations and South America (testified by the presence of some of these lineages in Venezuela [A2p3] or Colombia [A2w] or in Peru [A2ar]). The data cannot disregard the possibility of migrations from South America to Central America and the Caribbean. There are previous evidences pointing to this possibility [21,30,65] but the magnitude of these migrations needs further investigation.

**Figure 6:**
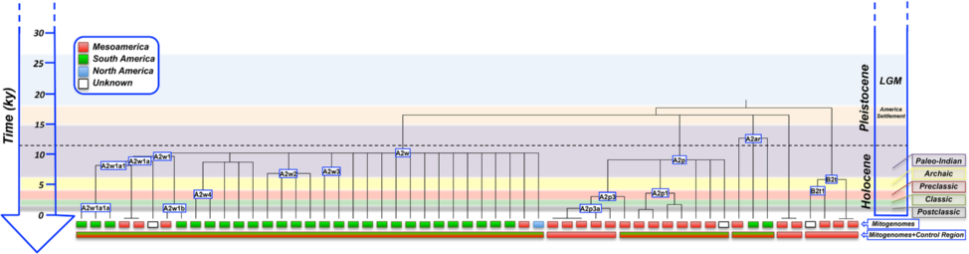
**ML phylogeny and TMRCA of the mitogenomes analyzed in the present study and Mesoamerican chronology.** The horizontal color rectangular boxes right below the tips of the phylogeny indicate the continental geographical location where the mitogenomes were sampled (as in Figures 4 and 5), while the color rectangular boxes in the bottom indicate the continental geographical location of the respective clades according to inferences carried out jointly on mitogenomes and control region data.

The phylogeographic characteristics of other mtDNA clades, however, point to demographic movements occurring to a more regional scale, almost exclusively within the Mesoamerican area. A number of these clades date back to the Pre-Classic and Classic Era (Figure 6); the development of the main Classic Maya Centers during the Classic period. This pattern can only be explained if a considerable gene flow across the different Maya territories is assumed.

Other population movements occurring during the Post-Classic Era (involving the Aztec, Mixtec, Totonac, Pipil, K’ich’e, Kaqchikel, among others) could also contribute to the dispersal of these lineages into this region. In particular, the role of the Nahua people [66,67], also referred in the literature to as Aztecs (Aztec civilization), could be particularly important as a source of more recent gene flow between Mexico and Guatemala. The Nahua received different denominations in different places. For instance, these groups were known as Pipiles in Guatemala, and their language was known to be a variant called Náhuatl Pipil. Various source of evidence (archaeological, linguistic, etc.) suggest that the Nahuas could have originated in the deserts of northern Mexico and southwestern USA and migrated into Central Mexico in several waves. Although the origin of the Nahua people is uncertain; it is well-known that the Nahua occupied the Mesoamerican territories ranging across modern-day Central Mexico to southwards in Central America in the XVI century, including Guatemala, El Salvador, Nicaragua, and even as far South as Panama [2]. The Pipiles were extinguished with the arrival of the Spaniards in colonial times, and the Nahua were gradually assimilated into ‘Mestizo’ society in most places. The last of the southern Nahua populations are the Pipil of El Salvador [68]. Some lineages found in Guatemala, such as haplogroup B1t1, are still found at high frequencies in present-day Nahua-speaking people from Mexico [31].

The large amount of shared variability observed between the different Maya ethnic groups (and with other Mesoamerican populations) analyzed in the present study and the lack of specific variability characterizing them is in agreement with a previous genetic study which obtained signs of genetic homogeneity among various Maya groupings by G-tests [37]. In contrast, these authors found significant heterogeneity from pair-wise comparisons between the Maya and other regional non-Mayan populations [37]. This would suggest that Mayan ethno-genesis is most likely very recent, perhaps occurring during the development of the Nahua civilization (1,100-500 ya). The large divergences observed in other cultural aspects of the Maya, such as linguistic ones, have probably developed very recently in the overall history of the Maya.

### Y-chromosomal SNP variation

The complete genotype results for Y-chromosomal SNP variation are given in Additional file 4. Haplogroup Q is the major branch on the Y-chromosome tree (89%) in the male Maya population set (Figure 2B). Q1a3a1(×Q1a3a1a-c) represents the most common haplogroup (81%), and 8% of the Y-haplotypes fall within Q (×Q1a3a1). The remaining subjects belong to the European haplogroups R1 (9%) and J2 (2%). The R1 sub-clades detected in Guatemalans were R1b1a2*(×xR1b1a2a1a1, R1b1a2a1a2a1b1a, R1b1a2a1a2b, R1b1a2a1a2c1a1a1), represented by three samples (two Q’eqchi’ and one ‘Ladino’), and one R1a1 member observed in one single K’iche’ individual. The J2 carrier self-describes as ‘Ladino’ and also reported two generations of ‘Ladino’ ancestry interrupted by a Q’eqchi’ maternal grandmother. J2 is the most common haplogroup in Europe [69].

The individual described above (#GT24) bearing the mtDNA Sub-Saharan haplotype L3b1a carries a Y-chromosomal haplogroup R1*(×R1a, R1b1) of European ancestry.

### Principal component analysis (PCA) and admixture analysis based on AIMs

**Figure 7:**
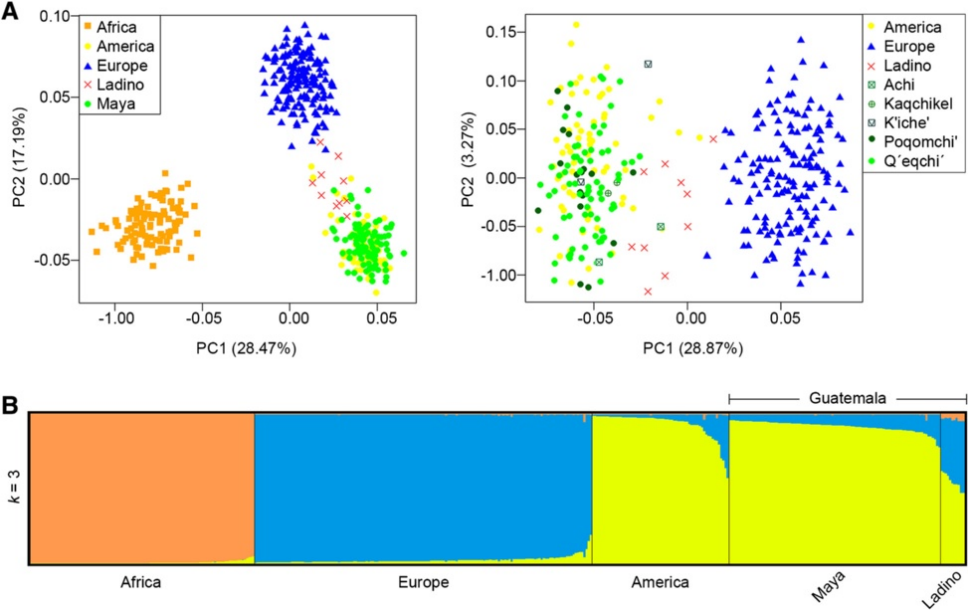
**PCA of Guatemalan profiles in different grouping schemes (A) and admixture analysis (B) based on the 46-AIM-InDels panel genotyped in the present study.** The PCA plot on the left considers the ‘Ladino’, the Maya (as a single group), and the reference data; while the PCA plot on the right shows the distinguish the Maya ethnic groups against the reference datasets **(A)**. For the admixture bar-plot **(B)** only results for the optimal *k* = 3 are shown.

PCA plot (Figure 7A) based on the 46 AIMs analyzed in the present study (Additional file 5) shows the relationship of the Guatemalan individuals with the three main CEPH panel continental groups, namely, Africans, Europeans and Native Americans, in the Euclidean space. The three reference continental populations show a clear differentiation (Figure 7A; left). PC1 (28%) separates Africans from non-Africans, while PC2 (17%) separates Europeans from the other two groups. Guatemalan Maya profiles all fall within the Native American cluster. Instead, ‘Ladino’ profiles form an scattered cluster located between Native Americans and Europeans; this pattern becomes clearer in a second PCA when eliminating the African reference samples (see PC1 [29%] in Figure 7A, right). The projection of the ‘Ladino’ profiles towards the European pole in the PCA mirrors a moderate European admixture in these individuals. On the other hand, there is no clear differentiation between different Maya ethnic groups.

Additional analysis carried out using Discriminant analysis of Principal Components (DAPC) underlines the outcomes of PCA and provides further assessment of between-population structures (Additional file 6).

The admixture bar-plot in Figure 7B indicates the ancestral membership for each individual in the three reference populations (African, European, and Native American) and the Guatemalan AIM profiles. Only the results for the optimal *k* = 3 are represented. These three components perfectly separate the profiles belonging to each of the main ancestral continental populations. The admixture bar-plot shows that most of the Guatemalan individuals have a dominant Native American ancestry (see also Figure 2C). However, a tiny portion of European co-ancestry at different scales can be observed across all Mayans. Therefore, admixture analysis agrees well with the results observed in PCA. Thus, for instance, those Guatemalan profiles with a higher European component correspond to those located close to the European cluster in the PCA (Figure 7A). Also consistent with previous analysis is the finding that no notable differences could be detected between the different ethnic Maya groups analyzed in this study. As expected, European co-ancestry is substantially higher in the ‘Ladino’ samples.

In contrast to the significant Native American and European ancestry of Guatemalans, the average African component is very low in Guatemala, and it appears almost exclusively in ‘Ladinos’ (3.6%). There was only one Maya who shows a moderate percentage of African co-ancestry (4.4%). This subject (#LaTinta_08, female) is of self-described Q’eqchi’ ancestry and carries a Native American mtDNA haplotype (B2t). This percentage of African ancestry in this Q’eqchi’ individual could simply mirror the variability of ancestry estimates using panels of AIMs containing a limited amount of SNPs [70], and not necessarily a real African genome ancestry.

Finally, for the AIM-InDel marker rs34122827, we found a third allelic state in one Mayan sample (#Marco_03). This allele corresponds to a T deletion occurring in the long allele background (allele 2D68Tdel). Interestingly, this variant was found to be specific to Europeans [71], whereas the carrier in our study is of K’iche’ ancestry.

## Conclusions

The results of uniparental *loci* show that the Maya population samples are mainly composed of Native American haplogroups with a minor presence of sub-Saharan (only on the mtDNA) and/or European lineages (only on the Y chromosome). AIM-InDels also points to the predominant Native American nature of the Maya (Figure 2C). In addition, ancestry proportions were different between ‘Ladinos’ and Mayans for the Native American and the European components, which is in agreement with previous studies [45].

In ‘Ladinos’, the main ancestry proportions are the Native American component (mtDNA: 91%; AIMs: 55%), and the European component in the male-specific genome (Y-chromosome: 75%). These results mirror the important demographic impact of the European colonizers in Guatemala (with a large effective population size) and their role in the extinction of the Native American population from the region. In particular, the patterns observed in ‘Ladinos’ indicate that the male population from Guatemala suffered more dramatically the consequences of the European conquest as mirrored by the differential ancestry components of the mtDNA and the Y-chromosome in the ‘Ladinos’. The Native American component of present-day Guatemalans was much better preserved in both male and female Maya, probably thanks to their geographic isolation in very inaccessible areas of the country.

Although African slaves arrived in Guatemala in the period between the VI and XVII century to replace the indigenous population as a labor force [72], our data indicate that the African genetic legacy in Guatemala is very low, and this agrees well with the documentation indicating the few amount of slaves arriving directly to the country. This is in contrast to other American populations, e.g. in Colombia [25], Brazil [73] and the Caribbean [63], but is in agreement with the patterns observed in El Salvador [26], which has no coast in the Caribbean (Guatemala has also limited contact with the Caribbean sea and even today, the country has difficult access through this coast). As shown by the admixture analysis based on AIMs, African ancestry is higher for ‘Ladinos’ (3.6%) than for the Maya (virtually 0%). The results as a whole are also in good agreement with the census: in modern-day Guatemala, ‘Afro-Guatemalan’ individuals comprise only ~1% of the total population and are found solely in a few communities living at the Caribbean coast where no subjects were recruited for this study.

Overall, the data reveal the existence of a fluid gene flow in Mesoamerica and a predominant unidirectional flow towards South America. The main movements could have occurred during the Pre-Classic (1800 BC-200 AD) and the Classic (200–1000 AD) Eras of the Mesoamerican chronology. This period coincide with development of the Maya, which was the most distinctive and advanced Mesoamerican civilization. Phylogenetic features of control region mtDNA data and the mitogenomes analyzed also suggest a demographic scenario that is compatible with moderate local endogamy and isolation in the Maya combined with episodes of gene exchange between ethnic groups. This pattern of variability is in agreement with a recent ethno-genesis of the Maya, which seems more established in cultural rather than a biological basis.

There is one main limitation in the present study. Thus, most of the demographic inferences carried out in this study are devoted to the analysis of the mtDNA variation (which only records the demographic processes affecting exclusively the female population). This is mainly due to the fact that the level of resolution provided by the mtDNA in our study is high compared to the resolution obtained for the other markers analyzed. Y-chromosomal and autosomal markers were genotyped in order to provide a more complete picture of the genetic patterns of Guatemalans. For instance, these markers have revealed the existence of an important gender-bias in this country (as it occurs in other American countries [74,75]), which moreover differs in ‘Ladinos’ and Maya.

Lastly, the data generated in the present study represent one of the very few genetic studies carried out in Native Guatemalans, and the ethnic groups sampled are analyzed here for the first time with a particular combination of uniparental and AIMs. The results provide new insight into the admixture characteristics of the Guatemalan population, with a clear gender bias observed in the ‘Ladinos’ but virtually absent in the Maya. The data also show important insights into the demography and the ethno-history of the Guatemalans and the important role of Mesoamerica as a passageway between North and South America. Last but not least, the data are also of particular interest from a forensic genetics point of view, as the results of our study may also contribute to the on-going work of the *Fundación de Antropología Forense de Guatemala* (FAFG) in prosecuting crimes against humanity that took place during the 1960–1996 civil war [43].

## Methods

### Sampling and DNA extraction

‘Ladino’ individuals were mainly recruited in cities (Guatemala Capital City and Cobán in the department of Alta Verapaz), while indigenous people were sampled directly in their communities in and around the highlands of Guatemala (Verapaz), the geographic heart of the country (Figure 1). Guatemala does not have legal regulations on the usage of native inhabitants DNA pool (according to the *Instituto Nacional de Ciencias Forenses* during the sample period; INACIF; http://www.inacif.gob.gt/). However, we ensured that every subject fully understood the aim of our study, which conforms to the Spanish Law for Biomedical Research (Law 14/2007-3 of July) and which was approved by the ethical commission of the Universidade de Santiago de Compostela. A document of informed consent was translated by a native Maya translator to the members of the villages and in particular to the donors. In case volunteers were analphabetic, fingerprints were used as signatures. The individual ethnic origin of participants was recorded by a detailed genealogy questionnaire. If self-reported family relationships were recognized during recruitment, just samples from one family member were considered for the analysis, independently from the degree of relationship. Distant relationships cannot be disregarded.

Recruitment of samples was limited by two main factors: (i) Maya linguistic diversity and their reservation towards medical study participation, and (ii) logistical difficulties for DNA sampling in a partially rough terrain with very difficult access.

DNA extraction was carried out from saliva samples on buccal swabs by organic standard procedures.

### Mitochondrial DNA sequencing and mtDNA SNP genotyping

A total of 110 samples (2 Achi, 2 Kaqchikel, 2 K’iche’, 11 ‘Ladino’, 18 Poqomchi’, and 75 Q’eqchi’) where analyzed for the mtDNA (see Additional file 1 for more details). All samples were amplified and double-strand sequenced for the entire mtDNA control region. Mutations are referenced with respect to the revised Cambridge Reference Sequence (rCRS) [76,77]. Haplogroup nomenclature follows Phylotree Build 16 (http://www.phylotree.org; [78]). The sequences were initially classified into haplogroups using *HaploGrep* [79] and manually checked according to recommendations [80]. Potential sequence artifacts were checked as reported previously [81-83]. In order to increase the phylogenetic resolution of mtDNA HVS-I/II within the Native American phylogeny, we genotyped coding region mtDNA SNPs (mtSNPs) using a single multiplex SNaPshot reaction, as described previously [64,84]. Unexpected mtSNP phylogenetic patterns according to the known phylogeny were confirmed by repeating the SNP genotyping using single-plex minisequencing and automatic sequencing.

Based on the information provided by the control region profiles (Additional file 1), 12 Native American lineages (carried by 10 Q’eqchi’ and 2 Poqomchi’) were selected for entire mtDNA genome sequencing following previously described protocols [27,85]; Additional file 2. The criterion for selection was mainly based on the particularities of the mutational changes carried by these profiles when compared against the known variability in other Native American datasets and phylogeny. The complete genomes analyzed in the present study have been submitted to GenBank under the accession numbers KM051465-KM051476.

### Y-chromosome SNP genotyping

A total of 58 males (1 Kaqchikel, 2 K’iche’, 4 ‘Ladino’, 8 Poqomchi’, and 43 Q’eqchi’) were genotyped for the Y-chromosome (Additional file 4) using a set of 26 SNP markers (see phylogeny in Additional file 7). Sixteen of these SNPs were analyzed in two reactions (Additional file 4) following the strategy of compound multiplexes described previously [86]. We adopted the revised haplogroup tree by the Y Chromosome Consortium YCC (2008) [87] and nomenclature adjustments according to the Y-DNA Haplogroup Tree 2013 by the International Society of Genetic Genealogy [88] (Additional file 7). We also applied two additional multiplex reaction containing SNPs M242, M3, M19, M194 and M199 (Additional file 4), which identify Native American populations as described before [89], as well as Y-SNPs M167, M222, U106, U198 and U152 (Additional file 4) belonging to the R1b1a2 haplogroup, in order to determine the most frequent European haplogroups.

### Genotyping of Ancestry Informative Markers (AIMs)

The same samples analyzed for mtDNA were also genotyped for 46 AIM-InDel markers [71], which allow the proportion of ancestry accounted for main continental groups to be estimated (Additional file 5). AIM-Indelplex PCR amplification and capillary electrophoresis were performed as described previously [28].

### Phylogenetic analysis and estimation of coalescent times

We used HVS-I data to build phylogenetic networks with the aid of the program Network 4.6.1.1 [90,91] and by hand. Hypervariable sites in HVS-I segment such as A16182C, A16183C, and T16519C were not considered (as usual).

Maximum parsimony trees were built for the complete genomes obtained in the present study and those collected from the literature belonging to haplogroups represented by the Guatemalan mitogenomes, and following the known worldwide phylogeny (Phylotree). Estimation of the coalescent times of the most recent common ancestor (TMRCA) was computed using two different procedures.

TMRCA was initially calculated using a ML procedure (Table 1). For this purpose, the software PAML 3.13 [92] was used assuming the HKY85 mutation model (ignoring indels, as usual) and using gamma-distributed rates (approximated by a discrete distribution with 32 categories) and three partitions: HVS-I (positions 16051–16400), HVS-II (positions 68–263), and the remainder.

TMRCA was also computed from the averaged distance (*ρ*) of the haplotypes of a clade to the respective root haplotype together with a heuristic estimate of the standard error (*σ*) calculated from an estimate of the genealogy (Additional file 3). These estimates were computed on the mitogenomes considering (i) the whole variation observed (excluding indels and hotspots) and (ii) using only synonymous mutations. The ‘star-likeness’ of the trees was measured using the star index *ρ*/*n* × *σ*^2^; this index can take values between 1/*n* (single haplotype representing *n* mtDNAs) and 1 (perfect star phylogeny) [23,93].

Both methods show very similar divergence ages when applied to mitogenomes. However, the averaged distance to the root shows an anomalous behavior on A2w1 and its sub-clades, with ages that are about twice (averaged on all sub-clades) larger than estimates based on ML (compare to a 1.2 of averaged discrepancy for the rest of the sub-clades). Estimates based on synonymous mutations show also large discrepancies with the ML method. In addition, A2w1 shows very low values of star-likeness (Additional file 3), which could be indicative of an overrepresentation of the A2w1 mitogenomes sampled in South America (coupled with the underrepresentation of A2w1 members from other Mesoamerican locations where this clade is probably present) or simply due to a limited sample size in this phylogenetic branch. Overall, the existence of a non-star-likeness phylogenetic pattern in A2w1 is what makes the ML method more reliable and consistent for the estimation of TMCRA. Thus, ML estimates were used for discussion throughout the text.

Mutational distances were converted into years using the corrected molecular clock proposed by Soares et al. [60].

### Statistical analysis

Admixture proportions from autosomal data were inferred by comparing genetic profiles from the present study with those publicly available from the Human Genome Diversity Cell Line Panel, HGCP-CEPH (Centre d’Etude du Polymorphisme Humain; [94]). These reference parental samples (*N* = 327) came from populations of three different continents: Africa (Central African Republic, Democratic Republic of Congo, Kenya, Namibia, Nigeria, Senegal, South Africa; *N* = 105), Europe (France, Italy, Orkney Islands, Russia, Russia Caucasus; *N* = 158), and America (Brazil, Colombia, Mexico; *N* = 64). Present-day East Asians were not taken into account as a reference population, assuming that these populations did not substantially contribute to the recent genetic heritage of the Guatemalan people, as is the case in other American locations [28,71,95].

Statistical analysis of AIMs included different tools aimed at disentangling the population structure of the Guatemalan study samples. Multivariate analyses were carried out using Principal Component Analysis (PCA). PCA condenses in a few principal components (usually two; PC1 and PC2) an initial set of data that can contain quantitative variables, into a group of fewer variables resulting in a linear combination of the originals.

PCA was performed using the statistic software R (R v.3.0.1, http://www.r-project.org/), together with the *SNPassoc* package (SNPassoc v.1.8-5, http://www.creal.cat/jrgonzalez/software.htm; [96]).

To further estimate individual ancestry proportions we used ADMIXTURE [97]. This software uses a ML estimation of individual ancestries from multilocus SNP data (AIMs).

Finally, phylogeographic searchers of mtDNA profiles were carried out on an in-house database containing >27,000 mitogenomes and >170,000 partial (mainly HVS-I) mtDNA sequences. Additional exploratory haplotype searchers were carried out on EMPOP (http://empop.org), Familytree (https://familysearch.org/), and the Sorenson (http://www.smgf.org/) databases. Note that frequencies obtained from these additional database searchers provide only approximate figures given that their web-interfaces were not conceived specifically for population genetic purposes (e.g. forensic casework in the case of EMPOP).

## Additional files

Additional file 1:
**Mitochondrial DNA hypervariable region sequencing and mtDNA SNP typing data for the Guatemalan samples analyzed in the present study.**
Additional file 2:
**Annotated variants of the mitogenomes analyzed in the present study and other mitogenomes used in Figures** 4 **and** 5 **.**
Additional file 3:
**TMRCA computed from the averaged distance (** ***ρ*** **) of the haplotypes of a clade to the respective root haplotype.**
Additional file 4:
**Y–chromosome SNP data for the Guatemalan samples analyzed in the present study.**
Additional file 5:
**Autosomes data for the Guatemalan samples analyzed in the present study.**
Additional file 6:
**Discriminant Analysis of Principal Components (DAPC) of Guatemalan samples carried out on AIMs.**
Additional file 7:
**Y chromosomal phylogenetic tree.** Polymorphism names are indicated above the lines (branches) and corresponding ‘rs’ numbers are shown below these lines. Bolded checkmarks (left) indicate haplogroups observed in the Guatemalan samples. The branch marked by M62 is now classified as private by the ISOGG consortium meaning that, according to the consortium “this SNP has not met the population distribution criteria for placement on the tree”.
