# Supplementary material for: Genomic insights on the ethno-history of the Maya and the ‘Ladinos’ from Guatemala
Source: BMC Genomics. 2015 Feb 25;16(1):131. doi: 10.1186/s12864-015-1339-1 (PMC4422311; doi:10.1186/s12864-015-1339-1)
Supplement: Additional file 3: — TMRCA computed from the averaged distance ( ρ ) of the haplotypes of a clade to the respective root haplotype. [file 12864_2015_1339_MOESM3_ESM.doc]

**Additional file 3**

Haplogroup coalescence time estimates based on the computation of the averaged distance of the haplotypes of a given clade to the respective root haplotype. Estimates were obtained for the complete genomes and considering only the synonymous variants. Estimate of the time (in kya) to the most recent common ancestor of each cluster, using evolutionary rate estimates in Soares et al. . First summand (*N*) refers to the complete mtDNA sequences displayed in **Figures 4** and **5** (see main text).

|  | **Entire genome** | | | | | | **Synonymous mutations** | | | | | |
| --- | --- | --- | --- | --- | --- | --- | --- | --- | --- | --- | --- | --- |
| **HG** | ***N*** | **Mean** | **95%CI** | **ρ** | **σ** | **ρ/(*n*×σ^2^)** | **N** | **Mean** | **95%CI** | **ρ** | **σ** | **ρ/(*n*×σ^2^)** |
| A2ar | 3 | 12.45 | 5.81-19.33 | 4.67 | 1.25 | 1.0 | 3 | 15.77 | 9.33-22.21 | 2 | 0.82 | 1.0 |
| A2p | 13 | 8.72 | 3.97-13.61 | 3.31 | 0.91 | 0.3 | 14 | 6.19 | 3.27-9.12 | 0.79 | 0.37 | 0.4 |
| A2p3 | 6 | 3.9 | 0-9.06 | 1.5 | 0.99 | 0.3 | 6 | 1.31 | 0-2.63 | 0.17 | 0.17 | 1.0 |
| A2p3a | 5 | 2.06 | 0-5.32 | 0.8 | 0.63 | 0.4 | 5 | 1.58 | 0-3.15 | 0.2 | 0.2 | 1.0 |
| A2p1 | 4 | 4.55 | 0.32-8.91 | 1.75 | 0.83 | 0.6 | 4 | 3.94 | 1.15-6.73 | 0.5 | 0.35 | 1.0 |
| A2p1b | 2 | 2.59 | 0-6.24 | 1 | 0.71 | 1.0 | 2 | 7.88 | 2.31-13.46 | 1 | 0.71 | 1.0 |
| A2p1a | 2 | 1.29 | 0-3.84 | 0.5 | 0.5 | 1.0 | 2 | − | − | − | − | − |
| A2w | 33 | 12.95 | 9.72-16.24 | 4.85 | 0.6 | 0.4 | 33 | 10.51 | 8.12-12.9 | 1.33 | 0.3 | 0.4 |
| A2w1 | 8 | 22.25 | 12.84-32.07 | 8.12 | 1.69 | 0.4 | 8 | 21.68 | 13.55-29.81 | 2.75 | 1.03 | 0.3 |
| A2w1a | 6 | 19.49 | 9.61-29.85 | 7.17 | 1.8 | 0.4 | 6 | 21.02 | 11.19-30.86 | 2.67 | 1.25 | 0.3 |
| A2w1a1 | 5 | 19.59 | 9.19-30.51 | 7.2 | 1.9 | 0.4 | 5 | 17.34 | 8.57-26.12 | 2.2 | 1.11 | 0.4 |
| A2w1b | 2 | 2.59 | 0-6.24 | 1 | 0.71 | 1.0 | 2 | − | − | − | − | − |
| A2w1a1a | 3 | 2.59 | 0-5.57 | 1 | 0.58 | 1.0 | 3 | − | − | − | − | − |
| A2w3 | 2 | 6.55 | 0.79-12.51 | 2.5 | 1.12 | 1.0 | 2 | 11.83 | 5-18.65 | 1.5 | 0.87 | 1.0 |
| A2w2 | 4 | 7.89 | 3.38-12.53 | 3 | 0.87 | 1.0 | 4 | 1.97 | 0-3.94 | 0.25 | 0.25 | 1.0 |
| A2w4 | 2 | 10.61 | 3.18-18.36 | 4 | 1.41 | 1.0 | 2 | 3.94 | 0-7.88 | 0.5 | 0.5 | 1.0 |
| B2t | 4 | 4.55 | 0.32-8.91 | 1.75 | 0.83 | 0.6 | 5 | 4.73 | 1.2-8.26 | 0.6 | 0.45 | 0.6 |
| B2t1 | 2 | 3.9 | 0-8.42 | 1.5 | 0.87 | 1.0 | 3 | 7.88 | 2.01-13.76 | 1 | 0.75 | 0.6 |

**References**

1. Soares P, Ermini L, Thomson N, Mormina M, Rito T, Rohl A, Salas A, Oppenheimer S, Macaulay V, Richards MB: **Correcting for purifying selection: an improved human mitochondrial molecular clock**. *Am J Hum Genet* 2009, **84**(6):740-759.
