# Supplementary material for: Genomic insights on the ethno-history of the Maya and the ‘Ladinos’ from Guatemala
Source: BMC Genomics. 2015 Feb 25;16(1):131. doi: 10.1186/s12864-015-1339-1 (PMC4422311; doi:10.1186/s12864-015-1339-1)

**Additional file 6**

**Discriminant Analysis of Principal Components (DAPC)**

While PCA is commonly used in human population genetic studies, DAPC is a relatively new methodology that has been recently developed . DAPC provides an efficient description of genetic clusters using a few synthetic variables called the discriminant functions. This multivariate analysis seeks linear combinations of the original variables (alleles), which shows differences between groups as best as possible while minimizing variation within clusters. Contrary to traditional methods such as PCA, which focus on the entire genetic variation, DAPC yields linear combinations of the original variables, which maximize differences between groups while minimizing variation within clusters. Based on the retained discriminant functions, the analysis derives probabilities for each individual of a membership in each of the different groups. This coefficient can be interpreted as “genetic proximity” of individuals to the different clusters. Therefore, DAPC aims to look for “discriminants” whose numeric values are able to separate the different classes defined from the cluster analysis, and from these values to classify new observations into those classes. Therefore, as for the Bayesian classification method, DAPC uses the concept of training set to create classification rules and a test set to gauge their efficiency. From a group of multivariate observations, already grouped into classes, DAPC discovers whether it is possible to create classification rules from the original observations (training set) that can reliably classify new observations (test set).

DAPC was carried out using the statistic software R v.3.0.1 (http://www.r-project.org/), together with the *adegenet* package (adegenet v.1.4-2, http://adegenet.r-forge.r-project.org/; ).

DAPC underlines the outcomes of PCA (see main document) and provides further assessment of between-population structures. DAPC scatterplots (**Figure 1** herein) show genetic clustering of Africans, Europeans, Native Americans, Ladinos and Mayas. We observed that little information is gained by adding PCs after the first ten (**Figure 1**, ‘PCA eigenvalue’ insets). We therefore retained 10 PCs (dark grey in **Figure 1** inset histograms) in each simulation to strengthen clustering and minimize the risk of over-fitting the discriminant functions. The insets of **Figure** **1** also show bar-plots of eigenvalues for the discriminant analysis of each analysis. We retained two discriminant functions (dark grey) in both analyses. As assumed when sub-grouping Guatemalan samples into ‘Ladinos’ and Mayas, it became clearer that ‘Ladino’ individuals are more closely related to Europeans than Mayas (**Figure 1A**). As with the PCA, sub-grouping of different Maya people did not lead to further sub-division (**Figure 1B**).

**Reference**

1. Jombart T, Devillard S, Balloux F: **Discriminant analysis of principal components: a new method for the analysis of genetically structured populations**. *BMC Genet* 2010, **11**:94.

**Figure 1**. DAPC of Guatemalan profiles based on the 46-AIM-InDels panel genotyped in the present study and considering the ‘Ladino’, the Maya (as a single group), and the reference data (**A**), and the different ethnic groups from Guatemala against the reference datasets (**B**). Clusters are shown by different colors and inertia ellipses, while symbols represent individuals. Smaller graph sets represent eigenvalues of DA and PCA.


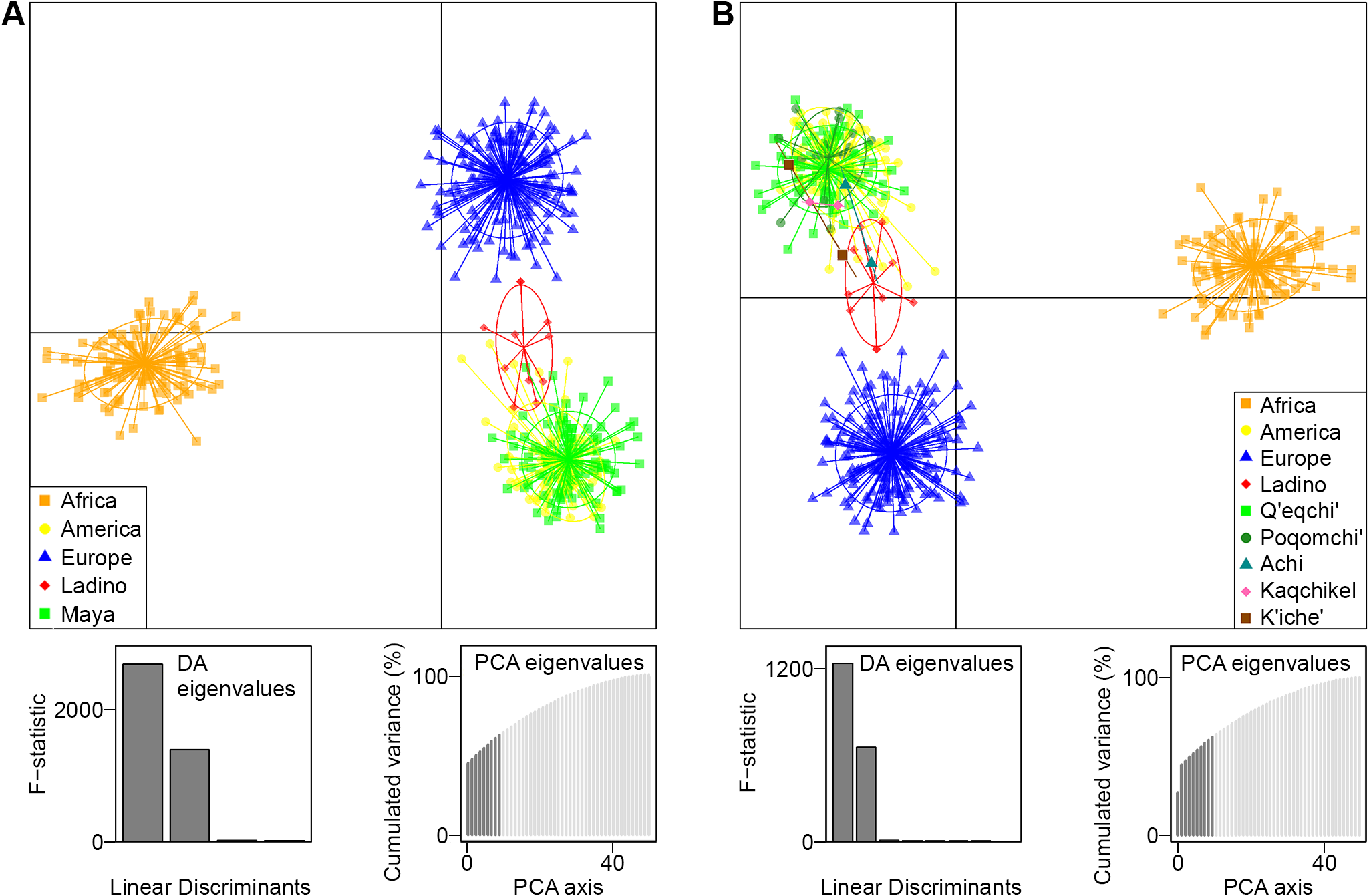

Supplement: Additional file 6: — Discriminant Analysis of Principal Components (DAPC) of Guatemalan samples carried out on AIMs. [file 12864_2015_1339_MOESM6_ESM.doc]
