# Supplementary figures and images for: Genomic insights on the ethno-history of the Maya and the ‘Ladinos’ from Guatemala
Source: BMC Genomics. 2015 Feb 25;16(1):131. doi: 10.1186/s12864-015-1339-1 (PMC4422311; doi:10.1186/s12864-015-1339-1)

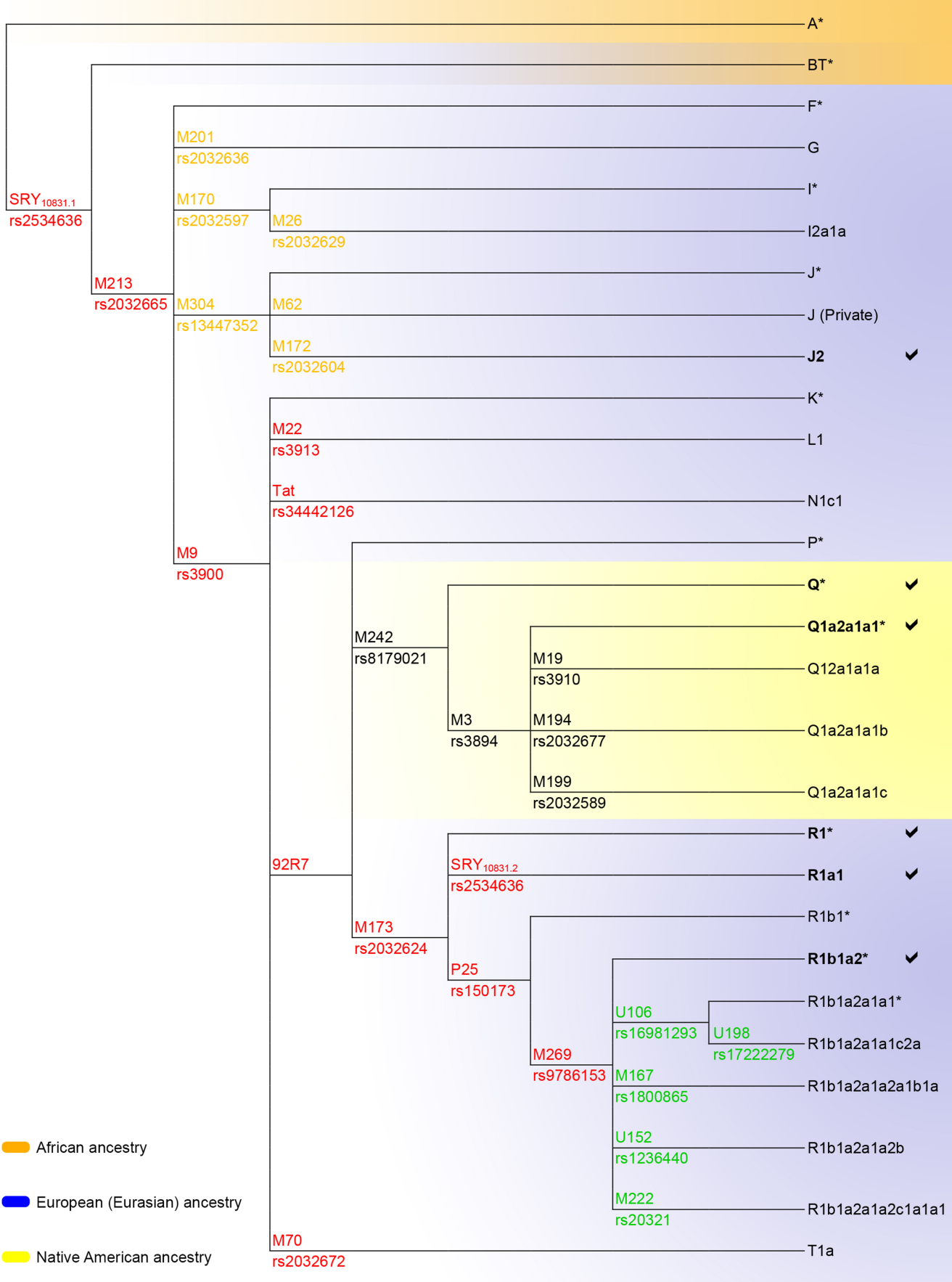

Supplement: Additional file 7: — Y chromosomal phylogenetic tree. Polymorphism names are indicated above the lines (branches) and corresponding ‘rs’ numbers are shown below these lines. Bolded checkmarks (left) indicate haplogroups observed in the Guatemalan samples. The branch marked by M62 is now classified as private by the ISOGG consortium meaning that, according to the consortium “this SNP has not met the population distribution criteria for placement on the tree”. [file 12864_2015_1339_MOESM7_ESM.pdf]
